# Polylactic Acid/Poly(vinylpyrrolidone) Co-Electrospun Fibrous Membrane as a Tunable Quercetin Delivery Platform for Diabetic Wounds

**DOI:** 10.3390/pharmaceutics15030805

**Published:** 2023-03-01

**Authors:** Francesca Di Cristo, Anna Valentino, Ilenia De Luca, Gianfranco Peluso, Irene Bonadies, Anna Di Salle, Anna Calarco

**Affiliations:** 1 Elleva Pharma s.r.l., Via Pietro Castellino, 111, 80131 Naples, Italy; 2 Research Institute on Terrestrial Ecosystems (IRET)—CNR, Via Pietro Castellino, 111, 80131 Naples, Italy; 3 Faculty of Medicine and Surgery, Saint Camillus International University of Health and Medical Sciences, Via di Sant’Alessandro, 8, 00131 Rome, Italy; 4 Institute of Polymers, Composites and Biomaterials (IPCB-CNR), Via Campi Flegrei, 34, 80078 Pozzuoli, Italy

**Keywords:** diabetic wound ulcer infections, quercetin, macrophage polarization, electrospinning, cytokines, polyvinilpirrolidone, polylactic acid

## Abstract

Diabetic wound infections (DWI) represent one of the most costly and disruptive complications in diabetic mellitus. The hyperglycemic state induces a persistent inflammation with immunological and biochemical impairments that promotes delayed wound healing processes and wound infection that often results in extended hospitalization and limb amputations. Currently, the available therapeutic options for the management of DWI are excruciating and expensive. Hence, it is essential to develop and improve DWI-specific therapies able to intervene on multiple fronts. Quercetin (QUE) exhibits excellent anti-inflammatory, antioxidant, antimicrobial and wound healing properties, which makes it a promising molecule for the management of diabetic wounds. In the present study, Poly-lactic acid/poly(vinylpyrrolidone) (PP) co-electrospun fibers loaded with QUE were developed. The results demonstrated a bimodal diameter distribution with contact angle starting from 120°/127° and go to 0° in less than 5 s indicating the hydrophilic nature of fabricated samples. The release QUE kinetics, analyzed in simulated wound fluid (SWF), revealed a strong initial burst release, followed by a constant and continuous QUE release. Moreover, QUE-loaded membranes present excellent antibiofilm and anti-inflammatory capacity and significantly reduce the gene expression of M1 markers tumor necrosis factor (TNF)-α, and IL-1β in differentiated macrophages. In conclusion, the results suggested that the prepared mats loaded with QUE could be a hopeful drug-delivery system for the effective treatment of diabetic wound infections.

## 1. Introduction

Nowadays, diabetic wound/foot ulcer infections (DWI), common multifactorial long-term complications in diabetes mellitus, represent a substantial economic burden to patients and the healthcare system due to a significant impact on patient’s health, quality of life, and life expectancy [1]. Patients suffering from diabetes experience metabolic disorders that affect the normal wound healing process. As a consequence, DWI may take a longer time to heal, leading sometimes, to amputation of limbs and often loss of life [2]. The hyperglycemic environment, typical of DWI, promotes bacterial colonization and biofilm formation, attending to abnormal immune function [3]. The proliferation of bacteria drives the wound into a long-lasting inflammatory phase, which induces neutrophils and macrophages to continuously produce inflammatory cytokines and reactive oxygen species (ROS), leading to the overexpression of metalloprotease (MMP-2 and MMP-9). MMPs secretion is responsible for extracellular matrix degradation, which impairs fibroblast adhesion and keratinocytes migration, resulting in slow wound healing [4,5,6,7]. Standard DWI treatment includes surgical debridement and dressings to facilitate a moist wound environment and exudate control. Moreover, due to the complexity of DWI pathophysiology, additional therapies such as negative pressure wound therapy, biological dressing, and hyperbaric oxygen treatment are recommended to achieve rapid wound healing [8,9,10]. In recent years, the use of natural-derived bioactive molecules has gained a significant increase in the management of DWI due to their low toxicity, and multiple pharmacological activities [11,12]. Among these, quercetin (QUE) due to its antioxidants, anti-bacterial/antibiofilm, and anti-inflammatory properties, represents an interesting therapeutic option to explore [13,14,15]. It is well-known, in fact, that QUE can improve common wound healing by increasing fibroblast proliferation, while decreasing fibrosis and scar formation, as well as ROS levels and bacterial adhesion and proliferation [16,17,18]. Nevertheless, the poor water solubility of QUE hampers its bioavailability, limiting the clinical application of this potent dietary bioflavonoid. Therefore, the development of nanotechnology-based strategies able to augment the local drug delivery of QUE represents a promising approach for better DWI management [19,20]. In this scenario, advanced nanofibrous dressings are essential to ensure a neutral and safe wound environment, achieving a better and faster wound closure. Thanks to the large surface area, small pore size, and gas permeability, nanofibrous electrospun membranes can simulate the structure of extracellular matrix, propelling and promoting cell proliferation, differentiation, and anti-bacterial effects [21,22]. Several polymers have been used for electrospun membrane manufacture, such as Poly lactic acid (PLA), Polyvinyl Alcohol (PVA), Poly-caprolactone (PCL), Polyethylene Oxide (PEO), and Polyvinylpyrrolidone (PVP) [23,24,25,26,27]. PLA is Food and Drug Administration (FDA)-approved synthetic polymer widely used for pharmaceutical and biomedical applications [28,29]. PVP is another non-toxic, biodegradable and fast-dissolving hydrophilic polymer highly applicable for the fabrication of drug-delivery systems [30,31,32]. Despite their proper characteristics, their applications in wound healing are restricted. Therefore, in the present study, the favorable properties of PLA-PVP co-electrospun nanofibers (PP mats) were combined with the biological activities of QUE. Recently, Zhou et al. fabricated a novel electrospun nanofiber membrane consisting of PCL, chitosan oligosaccharides (COS), and Quercetin/Rutin, with a good antioxidant and antibacterial activity, as promising wound dressings and drug delivery carriers for wound management [33]. Moreover, Gallelli and co-workers evaluated both the clinical efficacy and safety of hyaluronic acid nano-hydrogel embedded with QUE and oleic acid in the treatment of lower limb skin wound in 28 patients with diabetes mellitus [34]. The synthetized nano-hydrogels demonstrated a statistically significant reduction in the wound healing time without adverse effects. In another study, Jee et al. fabricated an enhanced topical delivery system featuring a combination of highly skin-permeable growth factors (GFs), QUE, and oxygen to accelerate re-epithelialization and granulation tissue formation in diabetic wounds [35]. 

In this work, the release kinetic of PP nanofibers loaded with three different concentrations of QUE (namely, PP/Q_5_, PP/Q_10_ and PP/Q_15_ mats) was analyzed in simulated wound fluid (SWF), miming the wound environment solution at human body temperature. The biological effect in terms of antioxidant and anti-inflammatory capacity of released QUE was also evaluated on an in vitro-induced inflammatory environment mimicking diabetic ulcers. Moreover, the effect of QUE on the macrophage switch from the M1 to M2 phenotype has been analyzed. To the best of our knowledge, a suitable local delivery platform that simultaneously elicits the QUE beneficial effect on inflammation, macrophage polarization and biofilm maturation has been rarely reported. The results presented herein suggest that the newly fabricated mats containing both hydrophobic and hydrophilic properties can be successfully used to meet the requirements of an ideal wound dressing in the treatment of DWI pathology. Indeed, the demonstrated biomimetic multifunctional features due to the presence of two unmixed polymers allow an ideal moist environment, simultaneously controlling biofilm formation at the wound site, promoting re-epithelization and tissue regeneration, eliminating, at the same time, the need for frequent dressing changes due to a sustained QUE release over a prolonged period of time. 

## 2. Materials and Methods 

### 2.1. Materials 

Polylactic acid (PLA) (Ingeo 4032D) with 0.7 mol% L-isomer, Mw = 2.1 × 10^5^ g mol^−1^ and the polydispersity (PDI) = 1.7 was supplied by NatureWorks LLC. Poly(vinyl pyrrolidone) K = 90 (PVP, average Mw = 360,000 Da) was purchased from Sigma-Aldrich Chemie GmbH (Schnelldorf, Germany). Chloroform (CHL), N,N-Dimethylformamide (DMF), ethanol (EtOH) and acetone with a purity ≥ 99.8%, Quercetin (QUE), Lipopolysaccharide (LPS, 8630), 3-(4,5-Dimethylthiazol-2-yl)-2,5-diphenyl tetrazolium bromide (MTT), phorbol 12-myristate 13-acetate (PMA, P8139), and IFN-γ (SRP3058) were purchased from Sigma-Aldrich (Milan, Italy). *Staphylococcus aureus* (ATCC 29213) and *Pseudomonas aeruginosa* PAO1 (ATCC^®^ BAA-47™) were purchased from the American Type Culture Collection (ATCC, Milan, Italy) and cultured following the ATCC’s guidelines. Human dermal fibroblasts (HDF) and human leukemia monocytic (THP-1) cell lines were obtained from ATCC and cultured following the ATCC’s guidelines. HDF were cultured in Dulbecco Modified Eagle Medium (DMEM) supplemented with 10% Fetal Bovine Serum (FBS), 2 mM L-glutamine, 100 IU/mL penicillin and 0.1 mg/mL streptomycin at 37 °C in 5% CO_2_ atmosphere. THP-1 were maintained in RPMI 1640 medium supplemented with 10% heat-inactivated FBS, 100 ng/mL of streptomycin, 100 U/mL of penicillin, and 2 mM L-glutamine. Mycoplasma testing was performed regularly to check for cell contamination. 

### 2.2. Preparation of PP/Qx Mats

To obtain PP/Qx mats, PLA and PVP solutions containing different amounts of QUE were prepared. Neat PLA solutions (coded as PLA) were prepared by dissolving 10% wt. PLA in chloroform/dimethylformamide (CHL/DMF, 80/20 *v*/*v*). Neat PVP solutions (coded as PVP) were prepared by dissolving 15% wt. PVP in ethanol. QUE loaded fibers were prepared with different amount of QUE with respect to the polymer mass (coded as PP/Q_5_, PP/Q_10_, and PP/Q_15_, respectively). First, QUE was dissolved in solvents, DMF for PLA (5% *w*/*w*–10% *w*/*w*–15% *w*/*w* respect to PLA) and EtOH for PVP (3.3% *w*/*w*–6.6% *w*/*w*–10% *w*/*w* respect to PVP); then the PLA polymer solution in chloroform and PVP powder were added to each QUE-based solution, respectively. All the solutions were stirred before use for at least 6 h. Each solution was loaded in a syringe and both of them were electrospun at the same time with NANON01 equipment (MECC Co., Ltd., Fukuoka, Japan), using a dual jet nozzle and a plate collector at room temperature and 10% relative humidity. After optimization of the process parameters for each solution, the flow rates for each solution were fixed at 2 mL h^−1^. The applied voltage and the distance between the dual jet nozzle and the collector, which was covered with aluminum foil, were adjusted to 25 kV and 30 cm, respectively, for both solutions to obtain defect-free fibers for further characterizations. To evaporate any residual solvent, electrospun fibers were kept under a fume hood for 24 h prior to characterization.

### 2.3. Physico-Chemical Characterization of the Membranes

Scanning Electron Microscope (SEM). The morphology of the membranes was evaluated using a FEI Phenom Desktop SEM (Eindhoven, The Netherlands). Before analysis, the samples were sputtered/coated with an Au-Pd alloy using a Baltech Med 020 Sputter Coater System and then mounted on aluminum stubs. The average fiber-diameter distribution was analyzed using ImageJ software (NIH, Bethesda, MD, USA).

By using energy-dispersive X-ray spectroscopy coupled with scanning electron microscopy (SEM-EDX), the atomic percentages were obtained.

Fourier-Transform Infrared Spectroscopy (FTIR). Chemical composition of membranes was investigated by means of FTIR coupled with attenuated total reflectance technique (ATR-FTIR). The spectra were acquired in the spectral region between 4000 and 400 cm^−1^. The analysis was performed using Origin software (Origin2020, OriginLab Corporation, Northampton, MA, USA). QUE spectrum was considered as positive control. 

Water contact angles (WCA). The water contact angles of the fibrous materials were measured using a FTA1000 (First Ten Angstroms, Inc., Newark, CA, USA) equipment. Drops of distilled water with a volume of 10 μL were deposited on the surface of the test specimens. The mean contact angle value was determined after averaging at least 5 measurements for each specimen. 

Stability test. The degradation study was determined by in vitro tests run in distilled water (pH 7). Small samples of PP and PP/Qx mats (5 mm × 5 mm) were immersed in 1.5 mL of medium and at different time intervals removed for analysis. Samples were kept under a fume hood for 24 h prior to morphological investigation by scanning electron microscopy.

Mechanical analysis. Mechanical properties of the obtained mats were analyzed by tensile tests performed at room temperature by using a 5564 Instron equipped with a 100 N cell load, at a crosshead speed of 5 mm/min. Tests were performed on 3 rectangular-style specimens cut from the electrospun mats. A micrometer screw gauge was used to determine the thickness of each sample (in the range between 30 and 40 µm).

### 2.4. In Vitro QUE Release

In vitro release of the QUE was investigated using static Franz diffusion cells (diffusion area 2.54 cm^2^, volume 12 mL) at 32 ± 0.5 °C to mimic skin surface temperature. Circular pieces of QUE loaded nanofiber mats (ø 1.6 cm) were mounted into a regenerated cellulose membrane (Spectra/Por^®^ MWCO 6–8 kDa, Spectrum Laboratories, Inc., Rancho Dominguez, CA, USA) and placed between the donor and receptor donor compartments. The receptor medium was simulated wound fluid (SWF, 2% BSA, 0.02 M calcium chloride, 0.4 M sodium chloride, 0.08 M tris(hydroxyl) aminomethane in deionized water, pH 7.5 adjusted using dilute HCl). At predetermined times, the sink conditions were met by replacing the amount of the solution removed from the cell by the fresh SWF of the same volume. The cumulative amount of QUE released in the medium was detected by HPLC-UV, as reported by Di cristo et al. [36]. Residual QUE was determined by immersing mats in chloroform/methanol solution (1:1 *v*/*v*). The amount of QUE was determined using HPLC-UV as reported before.

ChemStation software 4.03 27 January 2020 (Agilent Technologies, Milan, Italy) was used as System control and for data acquisition. Finally, the QUE release data were fitted to Korsmeyer–Peppas semi-empirical mathematical model, using Equation (1) [37,38]

(1) $$\frac{{\mathrm{QUE}}_{t}}{{\mathrm{QUE}}_{\infty }}=k\times t^{n}$$

where QUE*_t_*/QUE_∞_ is the fraction of QUE released at time *t*, *k* represents the release rate constant and consider the structural and geometric characteristics of the carrier, and *n* is the release exponent that indicates the drug-release mechanism.

### 2.5. Biofilm Analysis 

The effect PP/QUE membranes on biofilm formation were evaluated by crystal violet (CV) biofilm assay as described by Di Salle et al. with some modifications [39]. Briefly, mats with similar weight were sterilized by 15 min exposure to UV radiation and then covered with 750 μL of liquid medium broth containing *S. aureus* and *PAO1* (1 × 10^7^ CFU/mL). Bacteria were incubated at 37 °C in a humid atmosphere until 16 h. PP mat incubated in liquid medium broth was used as a negative control, whereas 750 μL of *PAO1* (1 × 10^7^ CFU/mL), and *S. aureus* (1 × 10^7^ CFU/mL), were used as positive controls. After 6, 12 and 24 h, the surface-adhered biofilm was gently washed with sterile PBS, air-dried for 30 min, stained with 0.1% *w*/*v* CV and finally dissolved in 96% ethanol. 

The absorbances at 570 nm (OD570) were measured using a microplate reader (Cytation 3, AHSI, Milan, Italy). 

Biofilm-viable bacterial cells were determined using the LIVE/DEAD^®^ Biofilm viability kit (Molecular Probes, Life Technologies Ltd., Milan, Italy), according to Bonadies et al. [40]. Images were acquired by using a Cytation 3 microscope (AHSI) equipped with a 10× objective. Viable bacteria emit green fluorescence (Ex/Em 510/530) due to the *MycoLight* Green fluorophore, whereas the dead ones, whose cellular membranes are damaged, incorporate propidium iodide and emit red fluorescence (Ex/Em 600/660). 

### 2.6. In Vitro Cell Studies

#### 2.6.1. Macrophages Polarization to M2 Phenotype

The PMA-differentiated THP-1 macrophages were obtained as described by Wang et al. [41]. Briefly, cells were seeded on plates or Petri dishes at 5 × 10^5^ cells/mL and incubated for 48 h with 50 ng/mL of phorbol 12-myristate 13-acetate (PMA, Sigma, Milan, Italy). Then, the PMA-differentiated THP-1 macrophages were washed with PBS and further treated with 100 ng/mL and 20 ng/mL IFN-γ (Sigma) for 4 h to induce M1 phenotype macrophages [42]. Then, M1 macrophages were cultured for 3 days in presence of PP/Qx conditioned medium. 

#### 2.6.2. Enzyme-Linked Immunoabsorbent Assay (ELISA)

Cytokines released in macrophages culture supernatant were quantified by ELISA technique according to Valentino et al. [43]. Antibody specific for IL-6, IL-12, or IL-10 were added to each well in 96-well plate and incubated at 4 °C overnight. Avidin conjugated Horseradish Peroxidase (HRP) solution was added into each well, incubated at room temperature for 30 min in orbital shaker and absorbance was measured by the Cytation 3 Microplate Reader at 450 nm.

#### 2.6.3. Real-Time Quantitative PCR (RT-qPCR)

Macrophage polarization and anti-inflammatory activity was evaluated by Real-Time Quantitative PCR (RT-qPCR) according to the manufacturer’s protocols. For RT-qPCR, total RNA was extracted from cells through TriFast (EuroClone, Milan, Italy), and cDNA was synthesized using Wonder RT cDNA synthesis Kit (EuroClone). Then, gene expressions of TNF-α, IL-1β, CCL18, CD206, IL-6, IL-12, and IL-10 were evaluated by 7900 HT fast Real-Time PCR System, (Applied Biosystem, Foster City, CA, USA) with SYBR Green PCR Master mix (EuroClone). Gene expression was quantified by the 2^−ΔΔCt^ method and normalized against 𝛽-actin used as the internal reference gene. Results were expressed as fold changes versus control. Primers used for RT-qPCR are reported in Table S1.

#### 2.6.4. Human Dermal Fibroblasts (HDFs) Proliferation and Migration Assays

To mimic the hyperglycemia environment, HDFs were cultured in DMEM supplemented with normal (5.5 mM) or high-glucose (25 mM) concentrations as reported by Sorooshian et al. [44], whereas cells cultured in 5.5 mM glucose-containing medium supplemented with 25 mM mannitol (as an osmotic control) were used as controls (CTL). The proliferation and migration of HDFs incubated with PP/Qx mats were investigated using the insert systems (Corning, Milan, Italy).

For proliferation, cells were incubated overnight in low-serum media (0.1% FCS) prior to conducting the experiment at 37 °C with 5% CO_2_. Then, dermal fibroblasts were subsequently exposed to media containing 5 mM or 25 mM glucose concentrations in presence of PP/Qx membrane for 24, 48 and 72 h. 3-(4,5-dimethylthiazol-2-yl)-2,5- diphenyltetrazolium bromide (MTT) assay was carried out as reported by Calarco et al. [45]. Cells Absorbance was measured at 570 nm using a microplate reader (Cytation 3; AHSI).

For scratch migration assay, cells were seeded on a 96-well plate (8 × 10^3^/well) and cultured to 90% confluence using the above-described protocol. Then, a standardized scratch wound was inflicted (T0) using sterile pipette tips (200 µL) [40]. Following PBS wash, cells were incubated with PP/Qx mats in 5 mM or 25 mM glucose containing medium for 24 h (T24). 0.1% mM mitomycin c (Merck Millipore, Milan, Italy) as a proliferation inhibitor was added. Fibroblast migration was photographed using an inverted phase-contrast microscope (Zeiss, Milan, Italy) and the percentage of wound closure was calculated according to the following equation:

(2) $$\mathrm{Wound}\;\mathrm{closure}\;\left(\%\right)=\frac{A_{0}-{\;A}_{t}}{A_{0}}\times 100$$

where A_0_ represent the wound area recorded at h 0 and A_t_ the wound area recorded at h 24 measured with ImageJ software. Experiment was conducted in triplicate.

### 2.7. Intracellular Antioxidant Activity

Total SOD-like and CAT activities were evaluated as reported by Valentino et al. [43] according to the manufacturer’s protocol. The mRNA levels of the oxidative markers superoxide dismutase 1 (SOD1) and catalase (CAT) were quantified by qRT-PCR as reported in Section 2.6.3.

### 2.8. Statistical Analysis

Student’s t-test was used for the quercetin release. For antimicrobial investigations assay, and quantitative real-time PCR, one-way analysis of variance (ANOVA) with Tukey’s post hoc test for statistical comparison were used. The difference was considered as statistically significant when *p* < 0.05. GraphPad Prism version 6.01 statistical software package (GraphPad, San Diego, CA, USA) was used to analyze all data. Results were expressed as mean ± standard deviation (SD).

## 3. Results and Discussion

### 3.1. Characterization of Electrospun Membranes

The use of electrospun fibers for drug delivery is widely investigated and one of the major challenges still remains to realize mats with tunable properties in order to have a more precise control of drug delivery and to focus on target applications [46]. Multi-spinneret electrospinning represents itself an excellent method to fabricate multiphase materials with specific and different characteristics such as degradation rate, mechanical properties, diffusivity and so on by utilizing the different properties of each component [47]. Du et al. prepared a bicomponent mat for wound healing by using PVA to keep moist environment promoting cell adhesion and proliferation and PCL to maintain structural integrity [48]. In another study, Scaffaro et al. reported the preparation and characterization of PCL/PLA co-mingled mats and their stability in three different buffered media (pH 4, pH 7 and pH 10): at the highest pH, the fastest degradation rate was observed, whereas in the alkaline medium, PLA was more sensitive than PCL [49].

Green tea extract (Cat) was incorporated using the double-nozzle electrospinning technique in gelatin (Gel)/PLA [50]. The authors demonstrated that the presence of Cat increased the diameter of fibers, whereas it decreased the contact angle of samples. Moreover, Cat elicits antibacterial activity toward *S. aureus* and *E. coli*, increasing the diameter of the inhibition zone. Finally, the hydrophilic nature of the Gel-Cat/PLA-Cat fibers improves the L929 fibroblast attachment, demonstrating a potential application of fabricated membranes for wound dressing. Our previous work demonstrated that QUE-loaded PLA nanofibers were able to counteract biofilm maturation in an oral acid environment (below 5.5) [40]. Moreover, it was demonstrated on an in vitro induced inflammatory model, that the released QUE showed a strong antioxidant and anti-inflammatory effect, suggesting that PLA-QUE fibers could be delivered into the periodontal pocket to simultaneously control inflammation and oral microbiome maturation. The results highlighted how PLA-QUE could be used as promising local adjuvants in periodontal disease. Despite the good properties demonstrated by our previous materials, wound dressing needs to maintain adequate dampness and a shield around the wound. For this reason, in the present work, PVP was chosen as the second polymer in addition to PLA to realize bi-component membranes because of its ideal properties such as inertness, chemical stability, no or low toxicity, lack of irritation to biological systems, biocompatibility and processability [51]. A hydrophilic nanofiber surface will be helpful for cells attachment and could provide a moist environment to accelerate drug permeation and the wound healing. However, a hydrophilic nanofiber surface may result in poor fiber stability. Therefore, the independent use of both polymers permits to obtain a bi-component mat with a sustainable degradation able to guarantee a different drug release behavior and improved membrane stability.

Hence, PP electrospun fibers loaded with different concentrations of QUE were prepared. 

**Figure 1:**
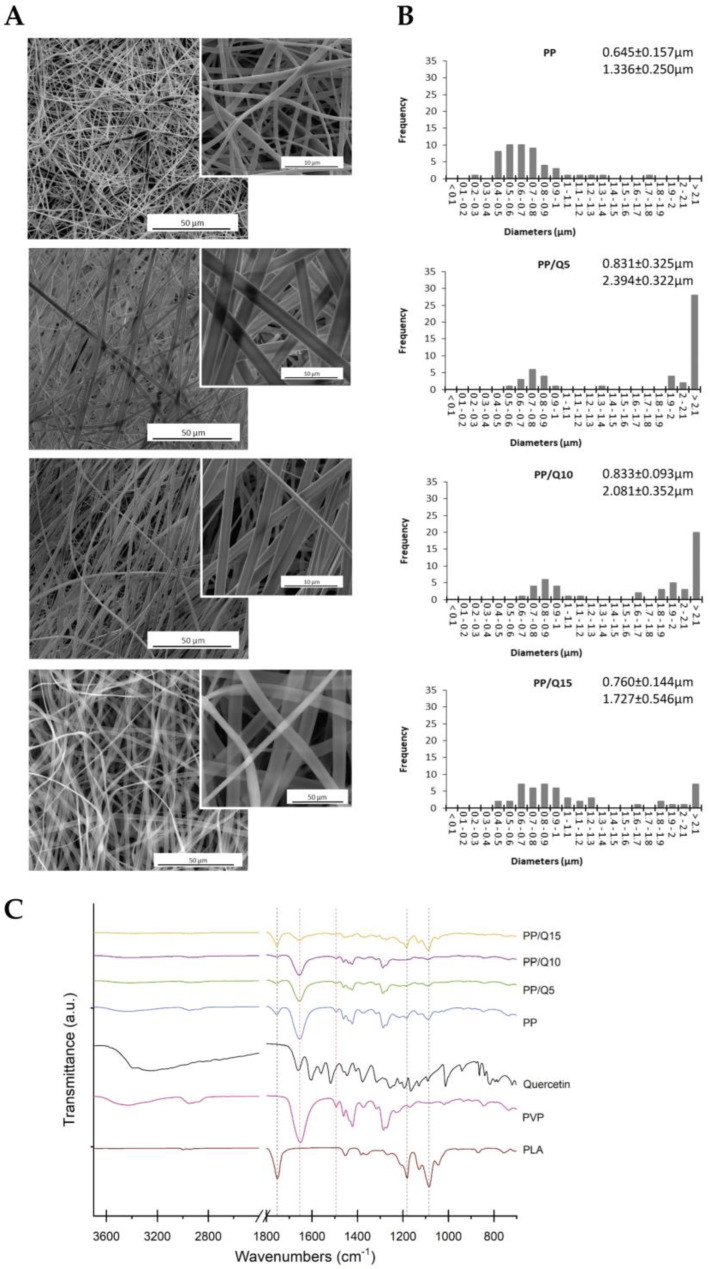
Characterization of the PLA-PVP/QUE membranes (PP/Qx): (**A**) SEM micrographs and (**B**) Fiber diameter distribution of PP, and PP at different QUE concentration (5, 10, and 15 coded as PP/Q_5_, PP/Q_10_, and PP/Q_15_, respectively). (**C**) FTIR-ATR spectra of quercetin, neat PLA, PVP, PP, PP/Q_5_, PP/Q_10_, and PP/Q_15_ fibers.

As reported in Figure 1, the micrographs of electrospun mat reveal defect-free fibers, uniform and quite homogenous in size for all their length except for PP sample that shows some beaded nanofibers. In particular, it is possible to observe a bimodal distribution of the diameter of fibers (one < 1 μm and another > 1 μm) related to the presence of two different fibers (one is PLA-based and the other is PVP-based) for each composition. As reported in our previous paper [30], the QUE-loaded PLA fibers have a diameter distribution centered at 0.4 ÷ 0.6 μm; for this reason, it is plausible to assume that the lowest diameters correspond to the PLA fibers, whereas the highest ones correspond to the PVP fibers. By adding QUE in PP samples, the morphologies of fibers became uniform and homogeneous in size above all for PP/Q_5_ and PP/Q_10_ samples. 

As expected, since the co-mingled mats are a microscopic physical mixture of non-interactive fibers, the EDX results (Figure S1) and the FTIR-ATR spectrum showed bands that are typical of all components. From the EDX analysis, the increasing amount of carbon with respect to oxygen by adding QUE is noticeable, whereas in the FTIR-ATR spectra, the presence of QUE is not detectable because the peaks of PLA and PVP and QUE overlap in the characteristic region (1500–1600 cm^−1^) (Figure 1C). However, the analysis proves the existence of a uniform co-mingled system as in the portion analyzed there are no zones with the presence of only one of the two polymeric phases.

The representative mechanical response of each sample (Figure S2A) exhibits a first linear segment where low deformation occurs due to the random and interlacing arrangement of the fibrous network. In fact, fibers act as an elastic material and undergoes uniform stretching throughout the length; thereafter, the formation of a neck region is noticeable; fibers breakage at the lateral side of the sample occurs (Figure S2B). This failure area enlarges with the strain due to the progressive breaking of the fibers until to failure. The mechanical behaviour recorded is similar for all compositions, by adding QUE the stress and deformation at peak slightly decrease and only at high additive concentration the stress at peak is reduced. These results can be explained considering a plasticizing effect of QUE; however, taking into account the composition and morphology of the mat, they require a more thorough investigation elsewhere.

### 3.2. Surface Wettability and Drug Release

Surface membrane wettability can affect the interaction between the biological fluids and the material surface [52]. Indeed, passage of the nutrients to the wound bed and membrane biocompatibility could be improved by material wettability. Furthermore, humid environment around the wound bed accelerates tissue restoration, alleviates pain and resists microbial attack [53]. 

**Figure 2:**
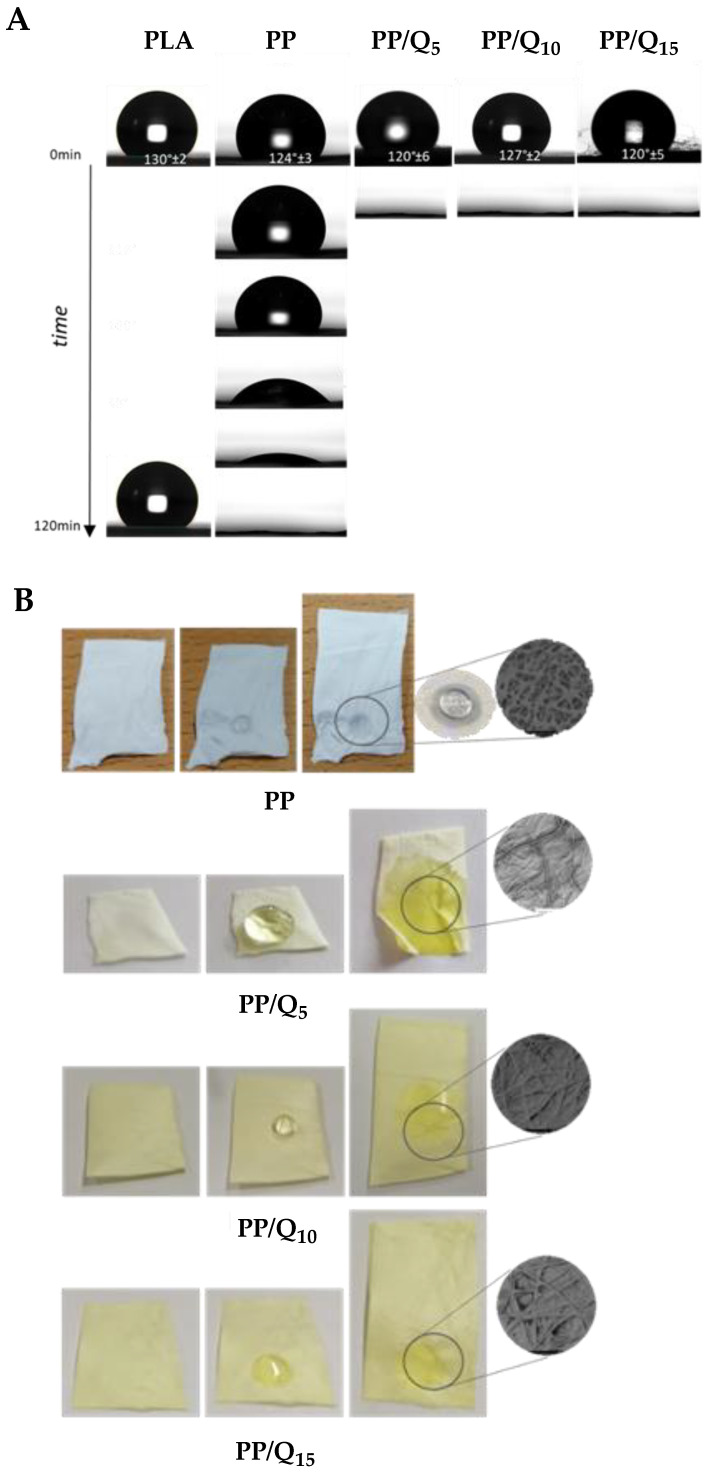
Behavior of synthetized membranes in contact with water: (**A**) water contact angle values of PLA, PP, PP/Q_5_, PP/Q_10_, and PP/Q_15_ samples; (**B**) visual inspection and morphological analysis of PP, PP/Q_5_, PP/Q_10_, and PP/Q_15_ surfaces.

The water contact angles (WCA) measurement confirmed the co-existence of the two different polymer matrices with opposite affinity for water (Figure 2A). It is well known that values of contact angles ≤ 90° are relate to hydrophilic surface, contact angles > 90° corresponding to hydrophobic surfaces and contact angle values >  150° are consistent to super-hydrophobic surfaces [54]. It is possible to observe for PLA neat membrane an initial WCA value of 130° ± 2° that did not change significantly by the time thus confirming a hydrophobic character. Whereas for PVP neat membrane the strong hydrophilic character is noticeable as soon the water drop is in contact with the surface since it is immediately absorbed. As PP membrane, the WCA value has an initial value of 124° ± 3° that goes to 0° in almost 2 h. The decreasing trend in water contact angles indicates that surface wettability of co-mingled mat appears as a hydrophobic one but by the time, it is strongly affected by the presence of PVP. On the contrary, for PP/Qx samples, WCA values start from 120°/127° and reach 0° in less than 5 s, indicating the hydrophilic nature of these samples. 

By the morphological analysis of wet surfaces (Figure 2B), after 60 s of contact with the water drop, it is possible to detect for PP samples the PLA fibers and the partially fusion of PVP fibers (Figure 2B). This behaviour is more evident for the PP/Q_15_ sample where, due to its solubility, the PVP phase cover the surrounding PLA fibers. This behavior is accountable to the presence of hydrophilic PVP and QUE affecting the overall comportment of the mat. In fact, the presence of quercetin inside the PLA matrix does not affect greatly the hydrophobic and stable behaviour of the membrane (WCA values of PLA/Qx membranes are in the range 120° ÷ 130°). The presence of PVP fibers inside the membrane, instead, reduces the WCA value by forming a compact phase surrounding PLA fibers and the additional presence of QUE amplify this behaviour due to its amphiphilic character [55].

Since wounds exhibit different extents of exudation, it is expected that wound healing process can be achieved by combining swelling, erosion and subsequent drug diffusion kinetics as part of the controlled drug release mechanism. In fact, most of the recently researched materials intended for wound dressings (either natural or synthetic) release incorporated drugs by a combined mechanism of either two or three above mentioned principles [56,57]. Based on the literature data, PLA has a low water absorption capacity, due to the large number of hydrophobic groups in the chemical structure, which develop strong intermolecular hydrogen bonds between the PLA molecules. Consequently, their availability to form hydrogen bonds with water ensures that the detachment of the PLA molecules rarely takes place and occurs very slowly. However, the addition of PVP, which contains a large number of oxygen functional groups, leads to increased polar groups. Interaction of water with the functional group of PVP conduces to disintegration of molecular chains of the polymer. This behaviour was investigated by a stability test in aqueous medium. The morphological analysis of mats after immersion in water at pH7 for different time intervals. (Figure S3) revealed that the PVP fibers disappear; in fact, a monomodal distribution of diameters is noticeable. Only small residues of PVP are visible among fibers. The residual fibers, corresponding to PLA, as confirmed by spectroscopy (Figure S4), kept their original morphology and homogenous surface without any evidence of deterioration. 

**Figure 3:**
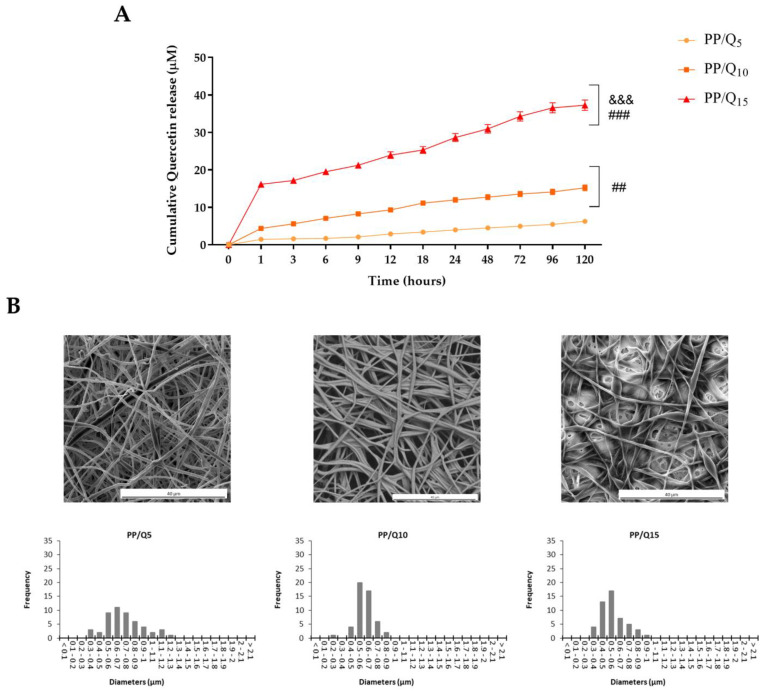
(**A**) Cumulative QUE release profiles at 37 °C of PP/Q_5_, PP/Q_10_ and PP/Q_15_. All samples were incubated for 120 h in SWF at pH 7.2. Six independent experiments were performed, and the results expressed as the mean of the values obtained (mean ± SD). Statistically significant variations: ## *p* < 0.01 and ### *p* < 0.001 versus PP/Q_5_, and &&& *p* < 0.001 versus PP/Q_5_ and PP/Q_10_. (**B**). SEM micrographs of PP/Q_5_, PP/Q_10_, and PP/Q_15_ fibers and corresponding diameter distribution after 6 h immersion in SWF.

In vitro drug release profiles of PP/Q_5_, PP/Q_10_ and PP/Q_15_ mats were evaluated in simulated wound fluid (SWF) at human body temperature. As depicted in Figure 3A, all samples exhibited a strong initial burst release, followed by a constant and continuous QUE release. In comparison to the previous reported mats loaded with QUE [40], the use of PVP accelerates the release rate in the initial stage mainly for the PP/Q_15_, followed by PP/Q_10_ and PP/Q_5_. In particular, PP/Q_15_ exhibited ~33% of QUE release in the first hour, corresponding to almost 16 µM, and reaching a plateau at the end of the experiment at ~37 µM (about 75% of QUE). 

As reported by Yu et al. [58] this behaviour can be attributed to the high PVP hydrophilicity. Indeed, the increase in the polymer–solvent interactions produce polymer matrix volume enhancement, which will ultimately result in the loosening of the polymer chains, causing the release of the bioactive molecules.

The reported release data of PP/Q_5_, PP/Q_10_ and PP/Q_15_ mats fitted the Korsmeyer–Peppas semi-empirical mathematical model, generally used to describe the drug release from polymeric nanofibers [59]. As showed in Table 1, a typical Fickian diffusion mechanism of QUE from PP fibers was identified by a value of the release exponent ≤ 0.45.

This kind of release kinetic is essential in wound healing treatment, to both reduce the possibility of biofilm maturation and progression and provide an initial QUE concentration sufficient to elicit antioxidant and anti-inflammatory effect. Then, the sustained release throughout the experimental period will provide a good environment over the long term to obtain a beneficial effect. 

The release profile is confirmed by membranes morphology after 6 h of immersion in SWF (Figure 3B). SEM observation reveals that although the membranes retain their fibrous structure, the fibers distribution is varied (monomodal distribution centered at lowest diameters), showing that the PVP is completely dissolved after immersion. 

### 3.3. Antibiofilm Activity

Diabetic wounds are susceptible to bacterial colonization, leading to systemic infection, which delays the wound healing process, resulting in prolonged inflammation [60]. To be an effective wound dressing, a nanofibrous mats have to efficiently protect the wound against the bacteria growth and infections, providing suitable wound breathing and also efficient handling of wound exudates and eventually accelerate the wound healing process [61].

**Figure 4:**
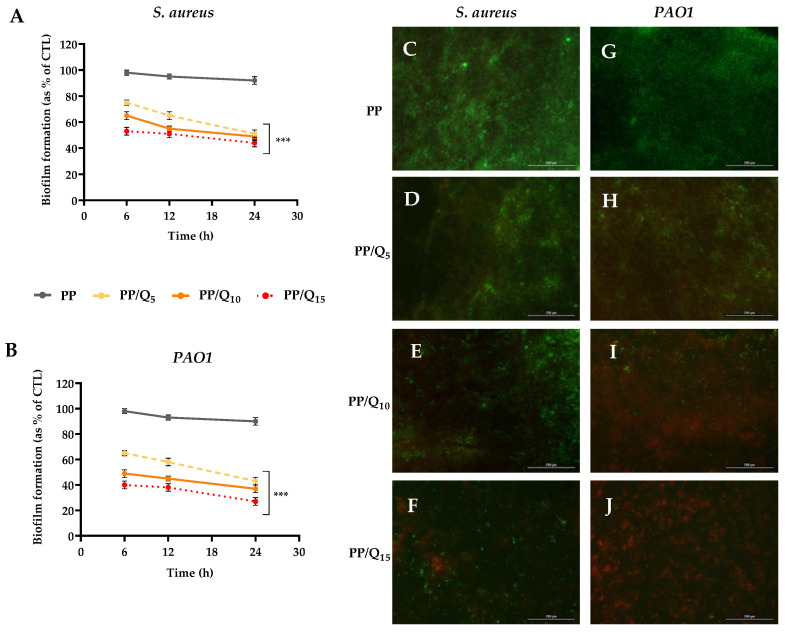
Antibiofilm activity of QUE-loading membranes. Biofilm formation was evaluated by CV assay, after 6, 12, and 24 h of incubation at 37 °C in presence of *S. aureus* (**A**,**B**) *PAO1* as described in the material and methods section. Biofilm formation was reported as a percentage in comparison with to the maximum amount of biofilm produced by *S. aureus* and *PAO1* grown (bacterial positive controls). For each sample, six different experiments were conducted, and the results expressed as the mean of the values obtained (mean ± SD). Statistically significant variations: *** *p* < 0.001 versus PP. (**C**–**J**) Fluorescent microscopy images of live/dead staining (scale bar 200µm, 40X magnification) of *S. aureus* (**C**–**F**) and *PAO1* (**G**–**J**) on PP (**C**,**G**), PP/Q_5_ (**D**,**H**), PP/Q_10_ (**E**,**I**) and PP/Q_15_ (**F**,**J**). Live bacteria were stained in green, and dead bacteria were stained in red. Live and dead bacteria in proximity resulted in yellow/orange color.

To ascertain the antibiofilm properties of PP/QUE membranes, the *S. aureus* and *P. aeruginosa* (*PAO1*) strains were used. These biofilm-producing bacteria play an important role in cutaneous chronic infected wounds. Two different biofilm analysis tests were used: a Crystal violet biofilm formation assay and a Live/Dead Bacterial Viability assay. Inhibition of biofilm development was assessed at different incubation times (6–12–24 h) using PAO1 and *S. aureus* bacteria in order to evaluate quercetin’s effectiveness. As shown in Figure 4, PP/Qx mats membranes inhibited the formation of biofilms in both bacterial strains in a dose-dependent manner. A significant reduction (*p* < 0.001) in biofilm formation was observed already after 6 h of incubation, in particular PP/Q_15_ membrane induced a reduction of about 47% and 60% in *PAO1* and *S. aureus*, respectively. Notably, after 24 h of incubation PP/Q_15_ strongly reduced biofilm development in both bacterial strain, exhibiting the maximum effect, with a reduction of about 56% against *PAO1,* and 73% against *S. aureus* (Figure 4A,B). These results are in line with the data of J. Ouyang et al. [62], which reported that QUE markedly impacted on *PAO1* biofilm formation at a concentration of 8–64 μg mL^−1^. Moreover, Jin-Hyung Lee et al. [63] reported the antibiofilm activity of QUE against three different strains of *S. aureus* already at a very low concentration (1 μg mL^−1^).

The susceptibility of *PAO1* and *S. aureus* to QUE-loaded mats was further evaluated via the Live/Dead BacLight Bacterial Viability Kit. This assay allows to differentiate between live and dead cells thanks to propidium iodide staining (red), able to selectively enters damaged bacteria membrane, whereas the fluorescent dye Syto9 (green) penetrates the membrane in both live and dead bacteria. As shown in Figure 4C,G, PP membranes permitted unperturbed biofilm formation, with a weak live/dead cell ratio, indicative of a bacterial population in stationary phase growth. Changes in viability of biofilm formed by *PAO1* (Figure 4D) and *S. aureus* (Figure 4H) were found with PP/Q_5_ membrane respects to PP. The biofilm formed by both bacterial strains on PP/Q_10_ membranes (Figure 4E,I) presents a decrease in biofilm mass/architecture, with a substantial proportion of dead cells. Notably, biofilm formed on PP/Q_15_ mat (Figure 4F,J) showed the highest live/dead cell ratio respect to PP membranes for both the microorganisms analyzed, with a drastically altered biofilm architecture. These qualitative findings confirmed the inhibitory effect of the new synthesized mats on the biofilm formation as well as their ability to induce bacteria cell membrane damage.

### 3.4. QUE-Loading Membranes Modulate the Macrophage Polarization

Wound healing is a very orderly and highly controlled process that requires the integration of complex cellular and molecular events characterized by distinct but overlapping phases: homeostasis, inflammation, cell proliferation, cell migration, angiogenesis and re-epithelialization [64]. During the initial phase, the role of macrophages is essential both to eliminate non-functional host cells and bacteria and create a favorable environment for tissue regeneration and repair [65]. Two distinct types of macrophages can be recognized: M1 macrophages, which function as pro-inflammatory mediators, and M2 macrophages, which act as natural feedback regulators from M1 macrophages. Based on their biological functions and phenotypes (secreted cytokines and surface markers), M2 can be further classified into four subtypes, i.e., M2a, M2b, M2c, and M2 [66,67]. M1-type activation is strictly related to ROS upregulation resulting in the production of pro-inflammatory cytokines (e.g., interleukin IL-6, tumor necrosis factor (TNF)-α, and IL-1β) [67]. Conversely, the activation of M2-polarized macrophages induces the secretion of anti-inflammatory cytokines, such as interleukin (IL)-10, and transforming growth factor-β (TGF-β), which leads to anti-inflammatory effects [68]. The macrophage M2a and M2c subtypes are both considered pro-healing and pro-remodeling. Their presence induces fibroblast and keratinocytes migration and proliferation, as well as the recruitment of endothelial stem cells leading to the development of granulation tissue and neovascularization. Therefore, in order to obtain a rapid wound healing process, the polarization of macrophages to anti-inflammatory M2 phenotype is necessary. Several studies report the potential activity of QUE in improving common wound healing by increasing fibroblast proliferation, while decreasing fibrosis and scar formation. Fu et al. showed that QUE was able to modulate the polarization of macrophages from M1 to M2 phenotype accelerating wound healing in the condition of diabetes [69]. In another study, Kim and coworkers demonstrated that QUE supplementation to high-fat-diet-fed C57BL/6 mice decreased the levels of inflammatory cytokines (TNFα, IL-6) and increased that of the anti-inflammatory cytokine (IL-10). Moreover, the hepatic inflammation reduction was accompanied by the upregulation of M2 macrophage marker genes (*Arg-1*, *Mrc1*) and downregulation of M1 macrophage marker genes (*TNF-α*, *iNOS*) [70]. 

**Figure 5:**
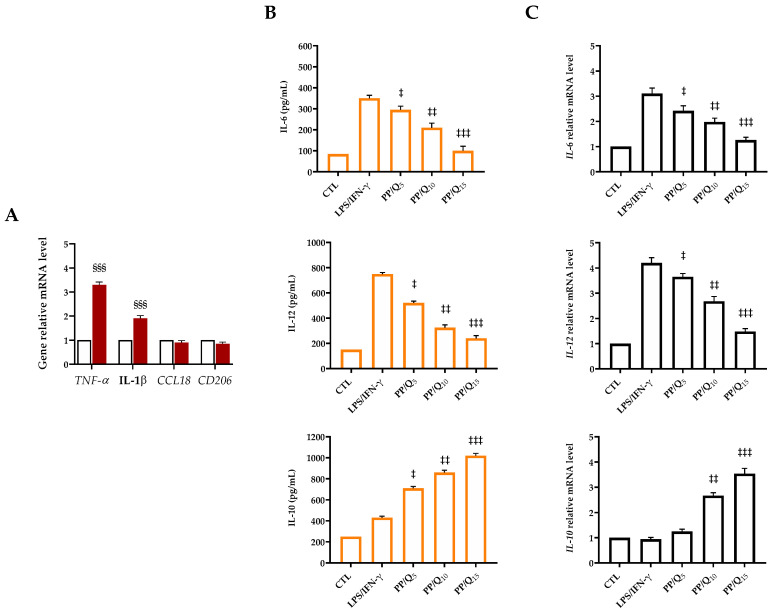
(**A**) mRNA expression of M1 macrophage markers after LPS/IFN-γ stimulation (10 pg/mL LPS + 20 ng/mL IFNγ) for 24 h analyzed by RT-qPCR. Stimulated macrophages were compared with unstimulated cells used as control (CTL). Data are represented as fold change over β-actin (2^−ΔΔCt^). Results are expressed as the mean of three independent experiments ± S.D (n = 3). Statistically significant variations §§§ *p* < 0.001 versus CTL. Inhibitory effects of QUE on the secretion (**B**) and gene expression (**C**) of inflammatory mediators IL-6, IL-12 and IL-10 in LPS/IFN-γ stimulated macrophages measured by ELISA assay and RT-qPCR. THP-1 cells were pre-treated with PP/Qx membranes for 24 h, then stimulated with LPS/IFN-γ for 24 h. PMA-differentiated THP-1 macrophages seeded on the culture plate without LPS/IFN-γ stimulation were used as control (CTL). Results are expressed as the mean of three independent experiments ± S.D (n = 3). Statistically significant variations ‡ *p* < 0.05, ‡‡ *p* < 0.01, and ‡‡‡ *p* < 0.001 versus LPS/IFN-γ.

In order to investigate the effect of PP/Qx on macrophage polarization, LPS/IFN-γ stimulation was used to mimic the biological microenvironment of body in responses to injury inducing M1 macrophage polarization [71]. As shown in Figure 5A, LPS/IFN-γ stimulated THP-1 cells strongly enhanced *TNF-α* and *IL-1β* expression (3.3- and 1.9-fold, respectively), whereas no significant expression of *CCL18* and *CD206* genes as M2 markers was detected, indicating the successfully polarization of the macrophages to M1 phenotype. ELISA results (Figure 5B) showed that the production of pro-inflammatory cytokines (IL-6 and IL-12) secreted from macrophages after LPS/INF-γ stimulation was significantly higher than that of the control group. As the QUE contents increased, a release reduction in these cytokines was observed in the cell culture supernatants, whereas the secretion of IL-4 and IL-10 increased. Accordingly, the expression levels of IL-6 and IL-12 in LPS/INF-γ stimulated cells were dramatically reduced in presence of PP/Qx membranes (relative to the housekeeping gene), whereas the anti-inflammatory factor IL-10 was significantly higher in QUE-treated groups than those in the control group (Figure 5C). As expected, the ability of QUE to modulate the anti-inflammatory response trigging macrophage polarization in the M2 phenotype was more evident in the presence of PP/Q_15_ with respect to PP/Q_10_ and PP/Q_5_. These results together suggest that PP/Qx membranes could promote macrophages polarization to M2 macrophages creating a microenvironment prone to promoting diabetic wound healing. 

With the purpose to obtain vascular tissue regeneration, Gui and colleagues manufactured a polycaprolactone (PCL) vascular graft incorporated with quercetin (PCL/QCT graft) [72]. In vitro studies demonstrate that released QUE was able to reduce the expressions of pro-inflammatory genes while increasing the expressions of anti-inflammatory genes in macrophages. Furthermore, the in vivo implantation in a model of rat abdominal aorta replacement led to the endothelial layer formation along the lumen of the vascular grafts at four weeks. More importantly, the presence of QUE stimulated the infiltration of a large amount of M2 phenotype macrophages into the grafts. Together, the reported data corroborated the hypothesis that the release of QUE may modulate the inflammatory microenvironment improving vascular tissue regeneration and remodeling in vascular grafts. Croitoru et al. discovered, via electrospinning, novel micro-scaffold matrices with triggered delivery capacity [73]. These fibrous scaffolds, based on PLA and graphene oxide (GO), were able to release QUE much faster (up to 8640 times compared with traditional drug-release approaches) when an appropriate electric field is applied. The QUE release from the PLA/GO matrix stimulated the production of IL-6 in fibroblast cells, which could be linked to an acute inflammatory response. 

### 3.5. QUE Restores HDF Cells Proliferation and Migration under Hyperglycemic Condition

Glucose-rich environment typical of diabetic status leads to a reduction in collagen synthesis, growth factor production, migration and proliferation of fibroblast and keratinocyte. Moreover, a continuous release of pro-inflammatory cytokines induces fibroblasts to secrete excess matrix metalloproteinases (MMPs) causing an imbalance between MMP (prevalently MMP-1 and MMP-8) and tissue inhibitory metallic proteinase (TIMP). This dysregulation results in the breakdown of collagen components and reduction in tissue mechanical strength [74]. In fact, dermal fibroblasts proliferate and migrate in the wound bed to produce collagen-rich matrix providing a scaffold for the migration of inflammatory cells [75,76]. Non-healing wounds associated with diabetes exhibit an interruption in the normal healing process. Several studies report that fibroblast cells isolated from diabetic foot ulcer patients showed impaired proliferation and migration suggesting dysregulated functional activity of fibroblasts in diabetic status [77]. Moreover, diabetes-derived dermal fibroblasts exhibited a reduced capacity to produce extracellular matrix proteins. In addition, the restoration of the epidermal barrier is compromised by the impair of keratinocytes proliferation and migration [78]. 

**Figure 6:**
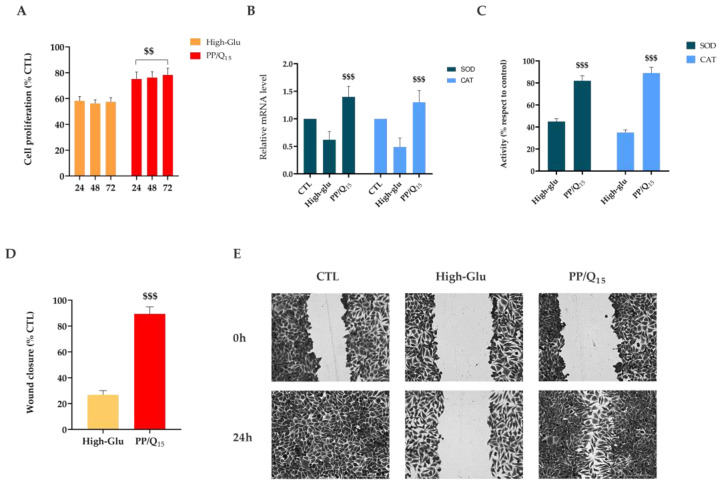
Effect of PP/Q_15_ membrane on fibroblasts migration, anti-oxidative genes expression and activities under hyperglicaemic conditions. (**A**) HDF proliferation after 24, 48, and 72 h in the presence of high glucose concentration (25 mM, high-glu) alone or with PP/Q_15_ membrane. (**B**) mRNA transcription level and (**C**) activities of Superoxide dismutase (*SOD*) and catalase (*CAT*) under hyperglicaemic conditions. (**D**) Quantitative analysis and (**E**). Representative images of wound closure at 0 and 24 h after treatment high concentration of glucose (25 mM, high-glu) alone or with PP/Q_15_ membrane. Scale bars are 50 μm. (n = 3). Results are expressed as the mean of three independent experiments ± S.D (n = 3). Statistically significant variations $$ *p* < 0.01 and $$$ *p* < 0.001 versus high-glu.

To mimic the hyperglycemic state typical of diabetes, dermal fibroblasts were cultured in media with a high concentration of glucose (25 mM). As shown in Figure 6A, fibroblast proliferation is dramatically reduced (about 1.3-fold) in high-glucose condition with respect to cells cultured in the presence of QUE at all points tested. The obtained results are in line with previous studies, which showed that free radicals generated by hyperglycemia delay cell replication time, triggering cell-cycle abnormalities independent of the osmotic mechanism [79,80,81,82]. 

Evidence suggests that antioxidants, such as QUE, might revert high glucose-impaired proliferation of HDFs possibly through a reduction in free radicals and activation of endogenous antioxidant systems via genetic modulation [83]. To confirm the involvement of ROS in the functional impairment of HDFs, the antioxidant effect of PP/Q_15_ was investigated. The expression of the anti-oxidative genes superoxide dismutase (SOD) and catalase (CAT) were analyzed by qRT-PCR. As shown in Figure 6B-C, high-glucose medium significantly induces a downregulation of both *SOD1* and *CAT* with respect to cells cultured in normal glucose-containing medium. Interestingly, pre-treatment with PP/Q_15_ results to a consistent increase in the expression of these genes, confirming ROS involvement in high glucose-induced impairments in HGFs. 

Wound-healing assay demonstrated that under the normal condition, the untreated cells migrate healing the wound after 24 h (Figure 6C). In contrast, the presence of high glucose concentration induced no cell migration toward the center with a significant reduction (*p* < 0.001) in wound closure. As expected, the cell migration rate under high-glucose concentration was accelerated in presence of PP/Q_15_ mat. In particular, released QUE promoted a significant (*p* < 0.001) migration of cells with a recovery of wound closure of about 89% respect to 26% of high-glu cells (Figure 6C). 

Mi et al. demonstrated that QUE obtained from *O. falcata* promotes both the proliferation and migration of fibroblasts, inhibiting pro-inflammatory cytokine secretion. Moreover, mice treated with QUE showed a restored dermal structure with high content of collagen fiber [84]. In another study, Irfan et al. showed a synergistic effect of human-umbilical-cord-derived MSCs and bioactive compounds of M. azedarach in treating cold burn wounds in both in vitro and in vivo wound models [85]. Preconditioned cells with 20 μM of QUE or Rutin, the main bioactive components of M. azedarach, enhance wound healing by reducing the inflammation, mitigating oxidative stress and enhancing neovascularization. Moreover, histological examinations revealed enhanced regeneration of skin layers along with hair follicles in the quercetin group. Taken together, the reported results reveal the ability of prepared mats to recover the capability of fibroblasts to migrate toward the wound and fill the gap, increasing the wound contraction also in the presence of hyperglycemic condition.

## 4. Conclusions

Impaired wound healing represents one of the most costly and disruptive complications in patients affected by diabetes mellitus, leading to extended hospitalization and limb amputations. In the present study, PLA-PVP-based mats loaded with quercetin were successfully prepared using the dual-jet electrospinning technique. The results of the SEM evaluation confirmed the formation of nanostructured and defect-free fibers. The fabricated fibers also exhibited a desirable wettability and biphasic QUE release with a rapid burst in the first hours, followed by a sustained release for a prolonged period. Antibiofilm studies showed that the mats prepared had good antibiofilm properties against PAO1 and *S. aureus*. Furthermore, the adopted formulation showed a significantly reduced toxic effect on dermal fibroblasts, and cell migration assays showed an increased migration of cells to the wound site. Moreover, released QUE significantly reduces the gene expression of M1 markers tumor necrosis factor (TNF)-α and IL-1β in differentiated macrophages, and limits pro-inflammatory cytokines secretion and gene expression. In addition, fabricated mats are able to repristinate the proliferation and migration properties of dermal fibroblasts cultured in hyperglycemic condition. Based on these results, it is possible to conclude that the developed mats could be a promising drug-delivery platform for the effective treatment of diabetic wound infections. 

## Figures and Tables

**Table 1:** Kinetic interpretation by using Korsmeyer–Peppas for QUE release from PP mats.

Mats	*k*	n	R^2^
PP/Q_5_	1.167	0.346	0.989
PP/Q_10_	4.984	0.238	0.988
PP/Q_15_	14.327	0.202	0.996

Legend: *k* is the release rate constant; n is the release exponent; and R^2^ is the correlation coefficient.

## Data Availability

The data presented in this study are available in the article.

## Supplementary Materials

The following supporting information can be downloaded at: https://www.mdpi.com/article/10.3390/pharmaceutics15030805/s1, Table S1. Primers used for RT-qPCR. Figure S1. Elements analysis by scanning electron microscopy-energy dispersive X-ray spectrometry (SEM-EDS) of PP and PP/Qx samples (All results in weight %). Figure S2. A. Stress-strain curves of PP/Qx electrospun mats. B. Image of a sample during tensile testing at different elongation percentages. Figure S3. Morphological analysis of mats after immersion test: diameter distribution (left) and SEM micrographs (right) at different time intervals. Figure S4. ATR spectra of mats before (PP, Quercetin, PLA) and after (PP/Q10) immersion test.
