# Supplementary material for: Polylactic Acid/Poly(vinylpyrrolidone) Co-Electrospun Fibrous Membrane as a Tunable Quercetin Delivery Platform for Diabetic Wounds
Source: Pharmaceutics. 2023 Mar 1;15(3):805. doi: 10.3390/pharmaceutics15030805 (PMC10054567; doi:10.3390/pharmaceutics15030805)

Article

Polylactic acid/poly(vinylpyrrolidone) co-electrospun fibrous membrane as a tunable quercetin delivery platform for diabetic wounds

Francesca Di Cristo [1,#], Anna Valentino [2,#], Ilenia De Luca [2], Gianfranco Peluso [2,3], Irene Bonadies [4,*], Anna Di Salle [2], and Anna Calarco [2,*]

[1] Elleva Pharma s.r.l. via P. Castellino, 111 – 80131 Naples, Italy
[2] Research Institute on Terrestrial Ecosystems (IRET)—CNR, Via Pietro Castellino 111, 80131 Naples, Italy
[3] UniCamillus, International Medical University, 00131 Rome, Italy
[4] Institute of Polymers, Composites and Biomaterials (IPCB-CNR) Via Campi Flegrei, 34, 80078 Pozzuoli (NA), Italy
* Correspondence: irene.bonadies@cnr.it (I.B.); anna.calarco@cnr.it (A.C.)
[#] These authors contributed equally to this work.

Table S1. Primers used for RT-qPCR.

Gene	*Accession Number*	*Forward (5′-3′)*	*Reverse (5′-3′)*
TNF-α	NM_000594.4	AACATCCAACCTTCCCAAACGC	TGGTCTCCAGATTCCAGATGTCAGG
IL-1β	NM_000576.3	TCCAGCTACGAATCTCCGAC	GCATCTTCCTCAGCTTGTCC
IL-6	NM_000600.5	CGCCTTCGGTCCAGTTGCC	GCCAGTGCCTCTTTGCTGCTTT
IL-10	NM_000572.3	TGTTTTCCCTGACCTCCCTC	GCTCCCTGGTTTCTCTTCCT
IL-12	NM_002187.3	CAGAGGGGACAACAAGGAGT	CTTGAGCTTGTGAACGGCAT
CCL18	NM_002988.4	TCAAGATGACGCTGCAATGC	CTTAGCCCAAAACCCAGCAC
CD206	NM_002438.4	ACCAGTTCCTTGACCTCAGG	ATATCGGAAAGGACTGCGGT
SOD	NM_000454.4	CCAGTGCAGGGCATCATCAA	TCTTCATCCTTTGGCCCACC
CAT	NM_001752.3	CGGACATGGTCTGGGACTTC	AACTGCCTCCCCATTTGCAT
ACTB	NM_001101.5	ACTCTTCCAGCCTTCCTTCC	CGTACAGGTCTTTGCGGATG

Figure S1. Elements analysis by scanning electron microscopy-energy dispersive X-ray spectrometry (SEM-EDX) of PP and PP/Q_x samples (All results in weight %).

**Spectrum PP**	**C**	**O**
Mean	54.96	45.04
Std. deviation	*1.13*	*1.13*

**Spectrum PP/Q_5**	**C**	**O**
Mean	61.00	39.00
Std. deviation	*0.87*	*0.87*

**Spectrum PP/Q_{10}**	**C**	**O**
Mean	73.63	26.37
Std. deviation	*0.47*	*0.47*

**Spectrum PP/Q_{15}**	**C**	**O**
Mean	68.24	31.76
Std. deviation	*1.19*	*1.19*

Figure S2. A. Stress-strain curves of PP/Qx electrospun mats. B. Image of a sample during tensile testing at different elongation percentages

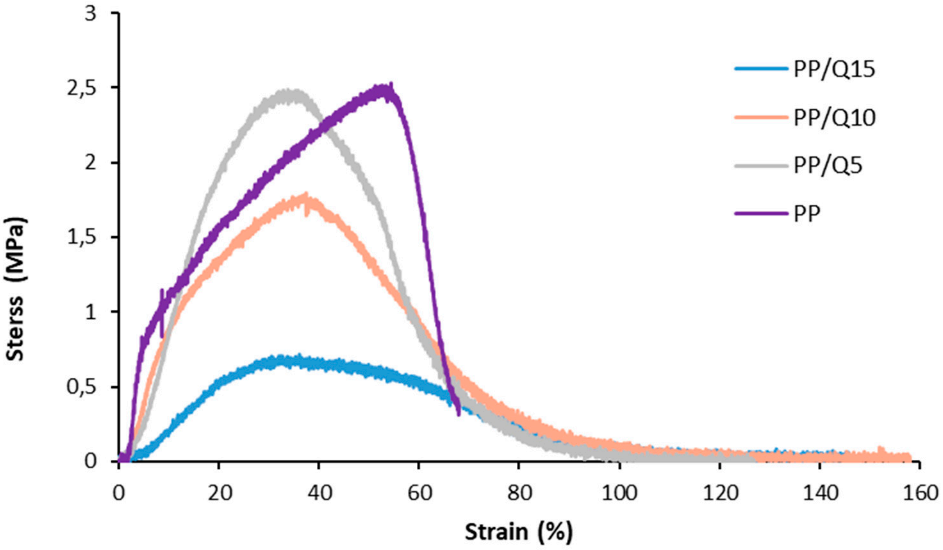

A

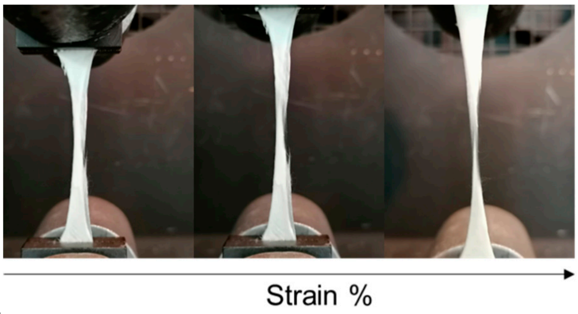

B

Figure S3. Morphological analysis of mats after immersion test: diameter distribution (left) and SEM micrographs (right) at different time intervals

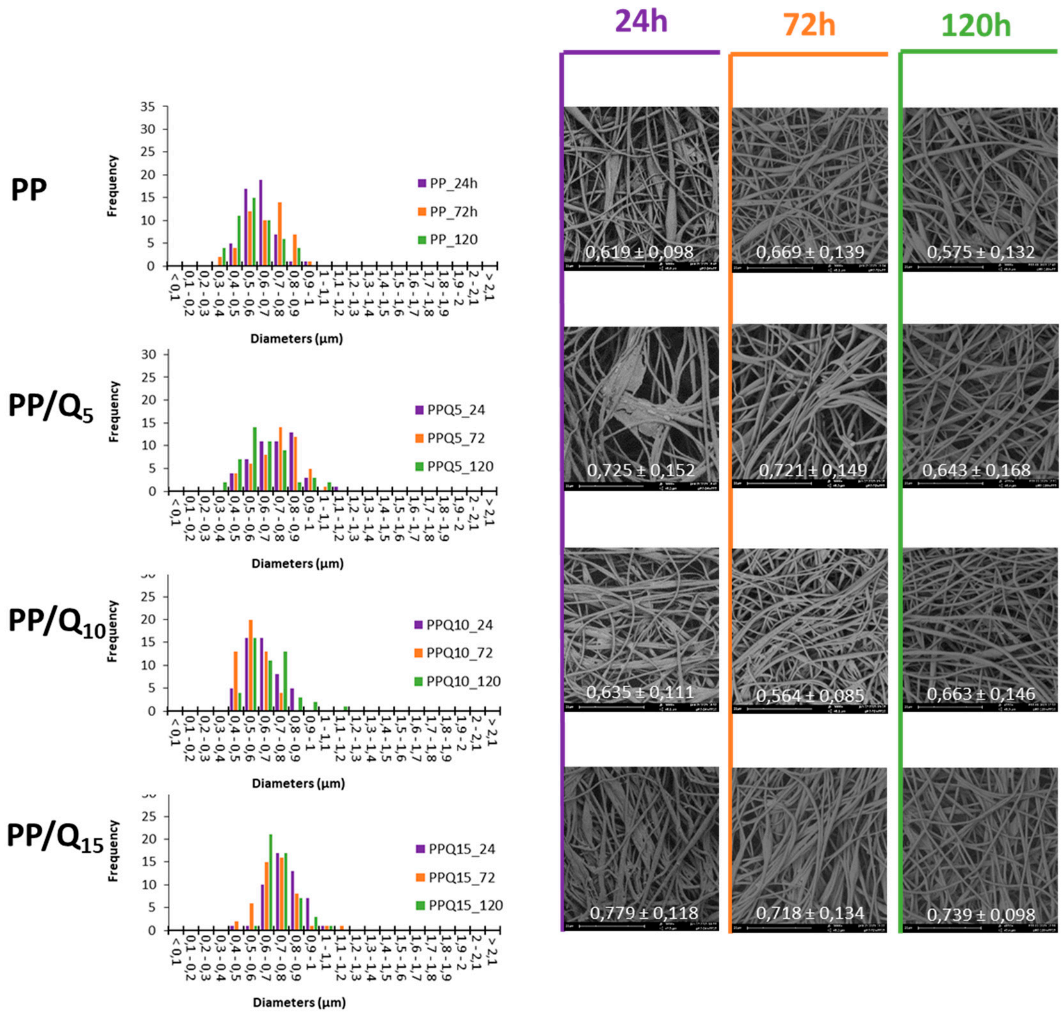

Figure S4. ATR spectra of mats before (PP, Quercetin, PLA) and after (PP/Q10) immersion test

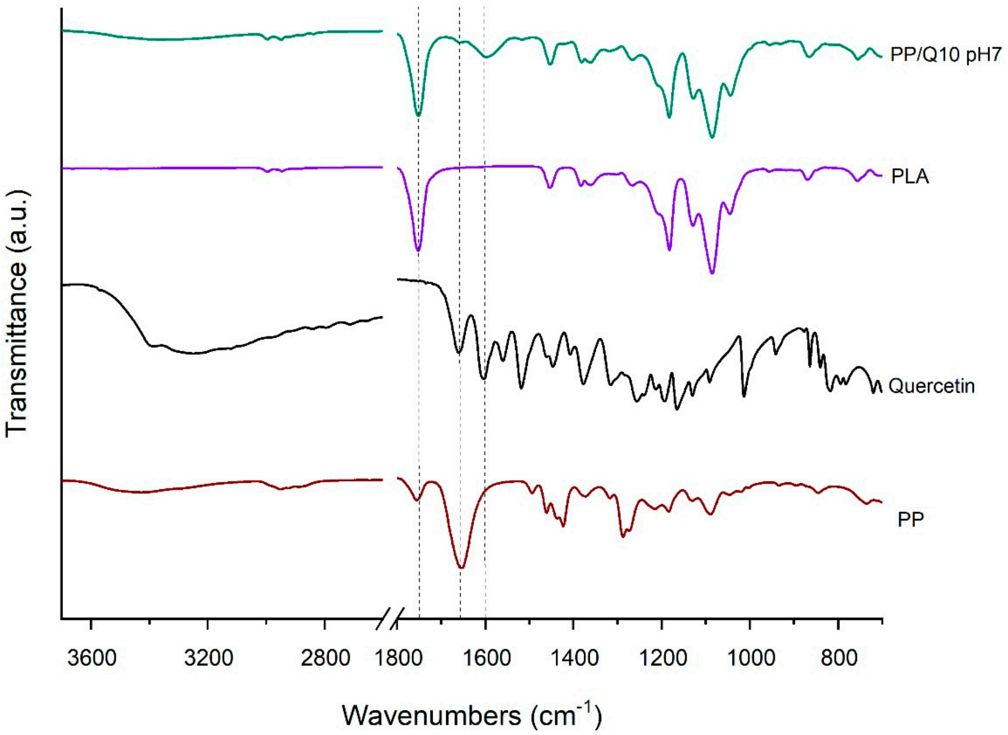

Supplement: Supplementary file 1 [file pharmaceutics-15-00805-s001.zip › pharmaceutics-2174151-supplementary.pdf]
